# An Emerging Field of Primate Social Neurophysiology: Current Developments

**DOI:** 10.1523/ENEURO.0295-17.2017

**Published:** 2017-10-04

**Authors:** Steve W. C. Chang

**Affiliations:** 1 Department of Psychology, Yale University, New Haven, CT 06520; 2 Department of Neuroscience, Yale University School of Medicine, New Haven, CT 06510; 3 Interdepartmental Neuroscience Program, Yale University School of Medicine, New Haven, CT 06510

**Keywords:** agency, allocentric representation, egocentric representation, nonhuman primates, social neurophysiology

## Abstract

Recently, there has been increased interest in investigating neurophysiological mechanisms underlying social interactions using a nonhuman primate model system. Several studies in this subfield, known as primate social neurophysiology, have begun to provide novel insights into how single neurons encode socially-relevant variables. This opinion piece intends to provide insight into the state of this field. In doing so, it discusses some common principles learned from primate social neurophysiology experiments.

## Significance Statement

Recently, there has been increased interest in investigating neurophysiological mechanisms underlying social interactions using a nonhuman primate model system. Several studies in this subfield, known as primate social neurophysiology, have begun to provide novel insights into how single neurons encode socially-relevant variables. This opinion piece intends to provide insight into the state of this field. In doing so, it discusses some common principles learned from primate social neurophysiology experiments.

## Introduction

Neurophysiologists are interested in discovering the neural codes that are used by the brain to represent variables from the sensory environments, and to plan and execute motor actions following wide-ranging cognitive operations. In the past decade or so, there has been an increasing trend in neurophysiological research to investigate the neurobiological mechanisms of social interaction. This is particularly true of nonhuman primate research, where exploiting the rich social behavioral repertoires of nonhuman primates has allowed researchers to focus on assessing how neurons from various brain regions signal information used for or as a consequence of social behavior. This research endeavor is often referred to as social neurophysiology. This opinion, by way of examining selected empirical results in rhesus macaques, discusses some common principles in social neurophysiology gained by neuronal recording experiments involving nonhuman primate social interactions. Specifically, this opinion narrates the knowledge gained so far indicating that social variables are represented in several parts of the brain involved in motivation and affective processing by way of agent-specific reference frames, contextual gain modulations, and, in some cases, privileged information processing, and mirroring.

Nonhuman and human primates share a vast array of social characteristics that are not found in other species. Macaques live in large, hierarchical groups, displaying extensive cohabitation and reciprocity (Mitani et al., 2012), and strategically acquire complex social information from facial expressions and the eyes (Emery et al., 1997). Not surprisingly, there are many similarities in the underlying neurobiology of social cognition between humans and macaques. As an example, in both humans (Mitchell et al., 2006; Burke et al., 2010; Apps and Ramnani, 2014; Sul et al., 2015) and macaques (Rudebeck, 2006; Yoshida et al., 2011; Chang et al., 2013b), the brain regions in the medial prefrontal and frontal cortices mediate social learning and prosocial behavior. As another example, the amygdala is particularly engaged when acquiring social information from facial expression as well as eye contact and gaze following in humans (Kawashima et al., 1999; Adolphs et al., 2005) as well as in macaques (Brothers et al., 1990; Gothard et al., 2007; Mosher et al., 2014). Furthermore, the value associated with particular social information is signaled by the orbitofrontal cortex (OFC) and the ventromedial prefrontal cortex in both humans (Hare et al., 2010; Smith et al., 2010) and in macaques (Watson and Platt, 2012). Overall, although monkeys and humans are undoubtedly different in many ways, studying social cognition in monkeys provide unique opportunities to investigate rudimentary neurophysiological mechanisms underlying human social behavior.

An increasingly acceptable view is that social processes in the brain are enabled by a variety of repurposed mechanisms that initially evolved to solve basic survival and reproductive needs (Chang et al., 2013a; Parkinson and Wheatley, 2015). Social events are closely associated with motivation and emotion. Not surprisingly, motivational and affective circuits in the primate brain are reliably engaged in computing social behavior (Behrens et al., 2009), sharing common fundamental operations (Joiner et al., 2017). Some of these brain regions include the anterior cingulate cortex (ACC), the OFC, the striatum, and the amygdala, all of which will be discussed later for their involvement in social interactions based on selected experiments in primate social neurophysiology.

## Agent-specific reference frames

Compared to more traditional neurophysiological research, social neurophysiology experiments frequently present novel problems in understanding neuronal encoding schemes across self and others. Debatably, the most interesting aspect of social neurophysiology research is in discovering how neurons involved in affective, motivational, sensory and motor processes encode information with respect to oneself and/or a conspecific. There is a parallel to this endeavor in the history of systems neuroscience research. Egocentric and allocentric representations in the brain have been recognized and studied in depth in the visuomotor and the spatial memory systems (Andersen et al., 1997; Burgess, 2006). Encoding variables in an egocentric frame of reference refers to cases when neuronal activity is tightly coupled to (i.e., systematically covaries with) one’s own body (e.g., body part position in space or on some location on the body). In contrast, an allocentric encoding refers to instances when neuronal activity is referenced to environmental location (e.g., world-centered) or objects (i.e., object-centered) that are outside one’s body. In the visuomotor system, a spatially-tuned neuron may encode a stimulus relative to one’s retina in an eye-centered or retinocentric frame of reference (Andersen et al., 1993), which would be classified as a type of egocentric representation. A different spatially-tuned neuron, in either the same or different brain area, may encode the position of the same stimulus relative to its environment but independent of one’s body, which would be classified as an allocentric representation. For example, in a paradigm involving body-under-head and body-plus-head rotations occurring with respect to a saccade target, visuomotor neurons in the monkey area 7a of the posterior parietal cortex modulate their responses according to the position of the head relative to the world (i.e., world-referenced, allocentric signal), but not the position of the head relative to the body (i.e., body-referenced, egocentric signal; Snyder et al., 1998). Similar egocentric and allocentric codes have been found for spatial memory (Feigenbaum and Rolls, 1991; Fyhn et al., 2004). For instance, place fields of the rat medial entorhinal cortical neurons near the postrhinal cortex display view-point-independent spatial information (i.e., allocentric; Fyhn et al., 2004). As in these examples from visuomotor processing and spatial memory, variables involved in social processing may also be encoded using distinct frames of reference (in this case, across agency) and that socially-relevant signals may undergo transformations to be represented in an egocentric or an allocentric manner (Chang, 2013). A need for such transformation processes is likely when social variables encoded in either an egocentric (e.g., relative to oneself) or allocentric (e.g., relative to a social partner) frames of reference need to be integrated to influence specific types of self- or other-regarding decisions.

**Figure 1.**
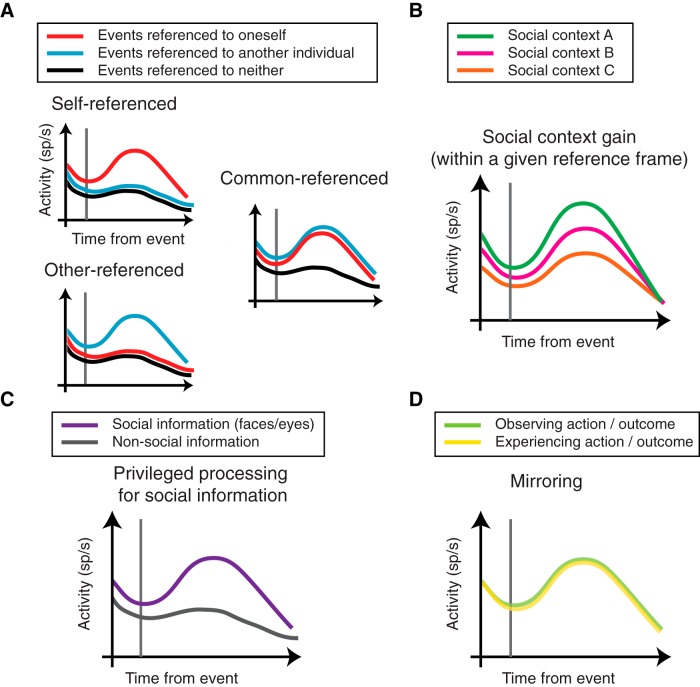
Schematic illustrations of the main neuronal principles discovered from primate social neurophysiology research. ***A***, Illustrations of three different neurons encoding task events in referenced to self (self-referenced), another individual (other-referenced), or both (common-referenced). ***B***, An illustration of a neuron whose activity is gain-modulated by different social contexts within a given reference frame. ***C***, An illustration of a neuron whose activity is specifically gated by particular social information, such as face and eyes. ***D***, An illustration of mirroring in a neuron with comparable firing rate profiles for experienced and observed actions and/or outcomes. Refer to the main texts for the empirical sources for these encoding schemes during social interactions.

A pioneering effort to understand how neurons represent self and other began when primate neurophysiologists examined how parietal neurons signal the location of food items differentially as a function of whether the location is shared by a conspecific (Fujii et al., 2007). Specifically, when two adjacent monkeys were not interacting for the food item, arm-motion related parietal neurons primarily signaled one’s own action. In contrast, when the food item was located on a shared, interactive space for the two monkeys, these neurons encoded complex patterns with respect to the actions of both self and other. More recently, a study under the framework of reward-guided decision-making examined the presence of agent-specific reference frames in three different structures in the prefrontal and frontal cortices. Specifically, this study compared the encoding of juice reward outcomes when a subject monkey made a decision to the delivery the reward only to himself, to a recipient monkey, or to neither. In OFC and the sulcus of ACC (ACCs), most neurons signal the reward outcome with respect to self. When the subject monkey makes decisions impacting another monkey, the firing rates of the majority of OFC neurons only encoded the reward outcome for the subject but not when the recipient monkey or neither was rewarded, whereas the firing rates of the majority of ACCs neurons only signaled when the subject monkey’s reward was omitted (i.e., when the reward went to the recipient or neither; Chang et al., 2013b). However, a group of neurons in the gyrus part of the ACC (ACCg) only encoded the reward outcome when the recipient monkey received the juice reward (allocentric reward coding; Chang et al., 2013b). Furthermore, in an experiment where a subject monkey was required to monitor another monkey’s action and outcome, a group of neurons in the medial frontal cortex (MFC) exclusively encoded the action of the other monkey (allocentric action encoding; Yoshida et al., 2011). In the same task, a large proportion of these MFC neurons specifically encoded the erroneous choices made by the social partner (Yoshida et al., 2012), suggesting that MFC harbors dedicated neurons for monitoring actions and errors by others. Similarly, when a monkey subject was taking turns with a human partner to constantly choose a new target located opposite from the target chosen by the human partner on the previous trial, many neurons in the lateral prefrontal cortex selectively encoded the target position of the monkey subject (egocentric encoding) or that of the human partner (allocentric encoding; Falcone et al., 2016). A further example of egocentric and allocentric encoding of social variables comes from an experiment where a monkey subject viewed a reward-predicting stimulus and touched it to realize the outcome concerning self and a conspecific. In this study, many neurons in the striatum preferentially signaled the reward outcome of self (egocentric reward encoding), regardless of whether the resulting action was performed by self or another monkey, whereas a smaller group of neurons encoded just the action of another monkey without encoding the reward outcome (allocentric action encoding; Báez-Mendoza et al., 2013). Furthermore, there are specializations with these striatal neurons for encoding erroneous performance. Specifically, although some striatal neurons signaled one’s own performance errors only, other striatal neurons signaled conspecific’s errors either exclusively or comparably to one’s own error (Báez-Mendoza and Schultz, 2016). Therefore, it is evident that both egocentric as well as allocentric encoding of social variables, which are anchored to the events of another individual, are present in the primate brain and are engaged during social interaction (Fig. 1*A*, three schematized neurons with different reference frames).

## Gain modulations by social contexts

In addition to the concept of agent-referenced signals, social information could be added to existing encoding schemes by way of gain changes in the signals, independent of reference frames used. In visuomotor processing, when the information about current eye positions (i.e., the location of the eyes relative to the head or orbital eye position) are added by way of multiplicative gain to a target position signal encoded in an eye-centered frame of reference, the signal now embodies new information about the target location with respect to the head, effectively creating a new representation (Andersen et al., 1985). Several findings in primate social neurophysiology support the idea that the information about a particular social variable may be added by either turning up or down the signal gain. Reward value gain-modulates the activity of predominantly egocentric OFC and ACCs neurons as well as mixed egocentric and allocentric ACCg neurons (Chang et al., 2013b). Furthermore, in an experiment where a subject monkey is required to lift a lever to deliver a reward to itself and to one of two conspecifics, the value tuning of individual OFC neurons are systematically gain-modulated as a function of the social hierarchical position of the conspecific which also receives reward (Azzi et al., 2012). Additionally, a recent study asked monkeys to play a prisoner’s dilemma (PD) task to examine neural signature of strategically cooperative decisions. In this monkey PD task, players could choose one of the two options (“cooperate” or “defect”) when the juice payoffs depend critically on the choices made by both monkeys, thereby encouraging strategic decisions taking into account the prediction of what the other player would do to maximize one’s reward. During this task, ACC neurons scale their activity to differentiate strategically predicted decisions made by a social partner from the current decision made by the subject monkey (Haroush and Williams, 2015). Finally, it has been shown that the differences between own and other’s rewards (inequity) are represented in the striatal neurons using gain modulations, equity (own reward = other’s reward), advantageous inequity (own reward > other’s reward), and disadvantageous inequity (other’s reward > own reward) are signaled via scaled firing rates (Báez-Mendoza et al., 2016). Taken together, in the prefrontal and frontal regions, a gain applied to either egocentric or allocentric signals may be used to combine social variables within existing representations (Fig. 1*B*, schematized neuron whose activity is gain-modulated by social context).

As alluded to by some examples above, motivational factors can exert a rather dramatic influence on firing characteristics of neurons recruited during social interaction. Motivational factors in social settings may differ as a function of impacting individuals with high versus low social status as well as making a self-generated versus observed outcome influencing other individuals. In the aforementioned experiment where a subject monkey is required to lift a lever to deliver a reward to itself and to one of two conspecifics, the reward value-dependent modulations in OFC neurons positively co-scaled with social context, defined by whether the reward was shared with a specific partner over another (Azzi et al., 2012). Moreover, when viewing the conspecific images with different values, more OFC neurons are modulated by social information conveyed in these images over juice magnitude (Watson and Platt, 2012). These findings suggest that the code for valuation in OFC neurons, during social settings, is strongly coupled to socially motivating factors. Furthermore, neurons in the basolateral amygdala (BLA) show correlated value tuning functions across self and other when an actor monkey makes social decisions about reward allocation across self and other, but this relationship is no longer present when the outcome is passively cued (Chang et al., 2015). These results suggest that the encoding schemes of BLA neurons markedly differ when motivation, or active decision-making, is involved.

## Privileged processing for specific social information

Finally, some social variables are highly salient over others to the primate brain, possibly having privileged access to the brain. As expected from the ecology of primate social behavior, the neural underpinnings of primate social behavior are tightly linked to facial expressions and social gaze interactions. Like in humans, faces convey special social information to monkeys. Recently, there has been a great amount of progress in how the primate brain computes facial information from both neurophysiological and neuroimaging studies in macaques. Face patches, first discovered in the temporal cortex, consist almost entirely of face-selective neurons (Tsao et al., 2006). A group of face patches form hierarchical social information processing streams, and are critical for face perception (Moeller et al., 2017; Sadagopan et al., 2017) as well as recognizing individual identity and familiarity (Chang and Tsao, 2017; Landi and Freiwald, 2017). Moreover, the activity patterns of face patch neurons are sensitive to contexts in which social interactions may take place (McMahon et al., 2015), and neurons within the face patches show diverse yet systematic interaction patterns with other brain regions (Park et al., 2017), revealing how face-related information is conveyed across the brain to guide social interactions. Taken together, face patches powerfully demonstrate the presence of privileged processing units for the most fundamental social information in primates that are sensitive to social settings. In addition, like humans, monkeys react to various facial expressions and adjust their future behaviors and follow gaze of a conspecific or make eye contacts to gain social information (Smuts et al., 1987; Ekman, 2015). Neuronal recording studies while monkeys view conspecifics’ facial expressions or the eyes have provided neural mechanistic insights into emotional processing and social gaze behavior. Recordings from the primate amygdala revealed that these neurons either increase or decrease their firing rates to encode either the facial expression or the identity of a conspecific, or some specific combination of both variables (Gothard et al., 2007), reflecting a signature of an integration process across identity and emotional expression. The amygdala neurons also embody a specialized group of neurons that are exclusively responsive to looking at the eyes of another individual or making direct eye contacts with others (Mosher et al., 2014). Furthermore, neurons in the amygdala have been shown to be involved in signaling either the production or monitoring of one’s own facial expression in reaction to viewing the face of a conspecific (Livneh et al., 2012). Signaling of facial expressions by the amygdala neurons is mediated by tactile neurons in the primate amygdala – these neurons show similar firing characteristics between self-produced facial expressions and those induced by tactile stimulation to the face (Mosher et al., 2016). Taken together, these findings suggest that certain, highly salient social information may recruit a specialized group of neurons that are specifically tuned to such ethologically-relevant information (Fig. 1*C*, schematized neuron with gated responses to particular social information).

## Mirroring signals

First discovered in the ventral premotor cortex (PMv or F5) of monkeys (Gallese et al., 1996), “mirroring” refers to when neuronal firing rates display similar characteristic patterns between one’s own movement and observing other’s movement. A series of studies that followed, in both humans and monkeys, have demonstrated the presence of mirroring by several brain regions in connection with social cognition (Gallese et al., 2004; Rizzolatti and Craighero, 2004). Such mirrored representations may serve unique functions in understanding other’s actions from the perspective of oneself. Mirroring, however, is not limited to the motor domain. In the social reward allocation task described earlier, a group of ACCg neurons signaled the reward outcome of self and other in an indistinguishable manner (Chang et al., 2013b) and the majority of BLA neurons showed correlated value tuning functions across self and other (Chang et al., 2015). Furthermore, as mentioned above, some striatal neurons encode performance errors regardless of whether the error was generated by self or other (Báez-Mendoza and Schultz, 2016). Therefore, mirror-type signals have been found across multiple functional domains, including movement, decision-making, and performance monitoring, and seems to be a common neural mechanism for encoding information in an agent-independent manner (Fig. 1*D*, schematized neuron whose activity mirrors experienced and observed actions/outcomes). How such agent-independent and aforementioned agent-specific neural signals interact to guide social behavior remains unknown.

## Concluding remarks

In summary, recent experiments in primate social neurophysiology suggest that social computations are conducted in the primate brain using agent-dependent reference frames, gain-modulated encoding of social and nonsocial variables, as well as the brain regions involved in motivation and affective processing and, in some cases, a specialized group of neurons tuned to highly salient social information. At this time, the field of primate social neurophysiology is quite young and often exploratory in nature given the lack of rich literature to guide testable hypotheses. There remain several core empirical questions for the field to answer within the next decade. For instance, it still remains unclear whether neurons distinguish between social variables and nonsocial variables sharing similar complexity. This is a particularly challenging question to satisfactorily address since the boundary between what we might call “social” and “nonsocial” processes, or “agency,” may not be categorically configured in the neural circuits, but rather lie on a continuum as a function of, for example, processing complexity and predictability. Furthermore, it would be particularly informative to know how and where in the brain distinct representations relating to self and other originate, merge or diverge during the social cognitive processing. The field of primate social neurophysiology may be able to provide new knowledge into how the human brain computes information about self and other, and makes decisions impacting fellow human beings. Advances in this endeavor may one day provide insight on how to improve the lives of individuals suffering from social dysfunction.
